# Benchmarking electrophysiological models of human atrial myocytes

**DOI:** 10.3389/fphys.2012.00487

**Published:** 2013-01-04

**Authors:** Mathias Wilhelms, Hanne Hettmann, Mary M. Maleckar, Jussi T. Koivumäki, Olaf Dössel, Gunnar Seemann

**Affiliations:** ^1^Institute of Biomedical Engineering, Karlsruhe Institute of TechnologyKarlsruhe, Germany; ^2^Center for Biomedical Computing, Simula Research LaboratoryLysaker, Norway; ^3^Center for Cardiological Innovation, Oslo University HospitalOslo, Norway

**Keywords:** cardiac modeling, atrial electrophysiology, atrial fibrillation, long term stability, restitution properties

## Abstract

Mathematical modeling of cardiac electrophysiology is an insightful method to investigate the underlying mechanisms responsible for arrhythmias such as atrial fibrillation (AF). In past years, five models of human atrial electrophysiology with different formulations of ionic currents, and consequently diverging properties, have been published. The aim of this work is to give an overview of strengths and weaknesses of these models depending on the purpose and the general requirements of simulations. Therefore, these models were systematically benchmarked with respect to general mathematical properties and their ability to reproduce certain electrophysiological phenomena, such as action potential (AP) alternans. To assess the models' ability to replicate modified properties of human myocytes and tissue in cardiac disease, electrical remodeling in chronic atrial fibrillation (cAF) was chosen as test case. The healthy and remodeled model variants were compared with experimental results in single-cell, 1D and 2D tissue simulations to investigate AP and restitution properties, as well as the initiation of reentrant circuits.

## 1. Introduction

Quantitative understanding of cardiac physiology and pathophysiology is of increasing importance, as the aging of the population predicts increasing prevalence of cardiac morbidity and mortality. Atrial fibrillation (AF) is the most common arrhythmia in clinical practice, with stroke being the major complication (Pedersen et al., 2006). Current drugs for AF treatment, however, have only moderate efficacy and may increase the risk of life-threatening arrhythmias (Ehrlich and Nattel, 2009). As a result, mathematical modeling and simulation of atrial electrophysiology as a supporting approach to AF investigation and treatment planning has garnered increasing interest in recent years. These investigative tools have been established in quantitative frameworks that can incorporate data from experimental studies, ranging from the level of the ion channel to the organ itself.

A multitude of models describing atrial cell electrophysiology have been developed over the last few decades for different mammalian species, including e.g., rabbit (Hilgemann and Noble, 1987; Lindblad et al., 1996) and canine (Ramirez et al., 2000). For human atrial cells, there have been two principal, longstanding models that reconstruct the action potential (AP) using ordinary differential equations (ODEs), based on overlapping experimental data: the Courtemanche et al. (1998) and the Nygren et al. (1998) models. In the absence of human data, both models rely partially on data obtained from other mammals, and have slightly different formulations of ionic currents, pumps, exchangers, etc., resulting in divergent behaviors (AP shape and restitution of AP duration) as reviewed previously by Nygren et al. (2001) and Cherry and Evans (2008).

While the usability of these two comprehensive cell models has been established, e.g., in consecutive studies of AF (Courtemanche et al., 1999; Zhang et al., 2005; Tsujimae et al., 2008), very little has been done to improve the physiological accuracy of the models, until recently. Maleckar et al. (2008) published a re-implementation of the Nygren model, with improved description of ion currents that underlie repolarization and rate dependence of the AP. Koivumäki et al. (2011) published the first cell model that accounts for the atria-specific spatiotemporal characteristics of the sarcoplasmic reticulum (SR) Ca^2+^ uptake and release, which further extended the Nygren and Maleckar models. Also Grandi et al. (2011) presented a novel model that utilized new experimental data to describe intracellular Ca^2+^ handling and introduced β-adrenergic and cholinergic regulation of cellular function to the regime of human atrial cell models. Furthermore, the Grandi model established a third line of pedigree in that this model is not based on either the Courtemanche or Nygren models, but rather on a human ventricular cell model, Grandi et al. (2010) published previously by the same group.

A short overview of these five human atrial cell models is given in Dössel et al. (2012), but as a comprehensive comparison of these has not been published previously, we aim to jointly characterize these models. In contrast to e.g., (Niederer et al., 2011) the accuracy or performance of the models should not be benchmarked. The goal of this study is to establish the principal characteristics and potential differences in the models in terms of (1) long-term stability; (2) the ability to reproduce alternans and AF-induced remodeling; (3) restitution properties in 1D tissue; and (4) dynamics of simulated arrhythmia in 2D. Furthermore, differences in calcium handling, AP morphology and computing times are investigated. These properties are quantitatively compared under the same simulation conditions for all models. Based on these analyses, we discuss the potential applicability of each model in addressing questions of particular relevance in cardiac electrophysiology. In addition, we briefly review the current limitations in model validation related to the availability and quality of published experimental data.

## 2. Materials and methods

### 2.1. Models of human atrial electrophysiology

**Figure 1:**
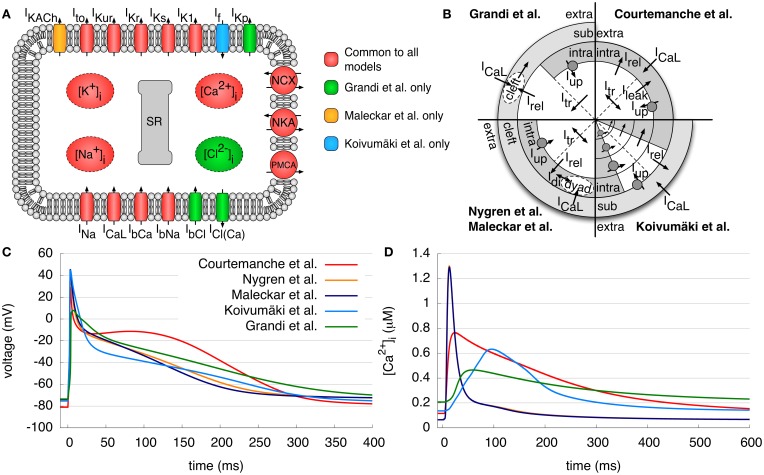
**Models of human atrial electrophysiology. (A)** Schematic of the cell membrane including the different modeled ionic currents and intracellular ion concentrations. **(B)** Schematic of the calcium handling with different compartments and currents of the models. **(C,D)** Resulting APs and the corresponding intracellular calcium concentrations after pacing for 50 s with a BCL of 1 s. Curves of N and M model calcium transient overlap. Detailed description of the figures and current abbreviations is given in section 2.1.

In this work, five different models of atrial electrophysiology were benchmarked. We will refer to the human atrial models specified in the following with the initial of the last name of the first author: C (Courtemanche et al., 1998), N (Nygren et al., 1998), M (Maleckar et al., 2008), K (Koivumäki et al., 2011), and G (Grandi et al., 2011). In all figures, the traces of the C model are red, those of the N model are orange, the M model dark blue, the K model light blue, and the G model green. An overview of the structure of the models, the differences in intracellular calcium handling, the resulting APs and the intracellular calcium concentrations are given in Figures 1A–D. Specific detail regarding the origin of experimental data used in model creation and validation and the resulting kinetic parameters of the current formulations is beyond the scope of this article and can be referenced in entirety in the original model publications.

The C model is based on the guinea pig ventricular model of Luo and Rudy (1994). Human experimental data was used to model the fast Na^+^ current *I*_Na_, the transient outward current *I*_to_, the ultrarapid delayed rectifier K^+^ current *I*_Kur_, the rapid and slow delayed rectifier K^+^ current *I*_Kr_ and *I*_Ks_, the inward rectifier K^+^ current *I*_K1_ and the L-type Ca^2+^ current *I*_CaL_. The Na^+^ and Ca^2+^ background currents *I*_bNa_ and *I*_bCa_, the Na^+^/Ca^2+^ exchange current *I*_NCX_, the Na^+^-K^+^ pump current *I*_NKA_, the sarcolemmal Ca^2+^ pump current *I*_PMCA_ and the intracellular calcium handling were based on a previous (canine) model. The SR is divided into two compartments, one for uptake and one for release of Ca^2+^. The Ca^2+^ uptake current *I*_up_ pumps Ca^2+^ into the SR and a leak current *I*_leak_ allows flow back into the intracellular space. The transfer current *I*_tr_ transports Ca^2+^ to the release compartment, where Ca^2+^ stores are emptied into the intracellular space by the Ca^2+^ release current *I*_rel_. For intracellular Ca^2+^ buffers, there are formulations for troponin, calmodulin and calsequestrin. A typical AP and the calcium transient (CaT) can be seen in Figures 1C,D, respectively. The AP shows a pronounced spike-and-dome morphology; the original application of the C model was the investigation of the rate dependence of the AP and its response to inhibition of *I*_CaL_ and *I*_NCX_.

The N model relies on almost the same human atrial data as the C model. Nevertheless, many of its formulations are initially based on the rabbit atrial model of Lindblad et al. (1996). The model employs the same transmembrane currents as the C model; however, *I*_Kur_ is denoted the “sustained outward K^+^ current *I*_sus_.” An additional electroneutral Na^+^ influx is incorporated to ensure long term stability of the intracellular ion concentrations. Furthermore, a cleft space surrounding the cell describes a limited extracellular volume permitting the accumulation or depletion of ions. In addition, the intracellular Ca^2+^ handling is modified by a description of the dyadic space between *I*_CaL_ channels and the SR, where the Ca^2+^ concentration may differ from the cytosolic concentration. As a result of these differences, the CaT has a higher amplitude and is much shorter (≈300 ms) than that of the C model (Figure 1D). Accordingly, the AP of the N model has a more triangular shape and lacks a dome as compared to the C model (Figure 1C). The first application of the N model was the investigation of differences between rabbit and human atrial repolarizing currents.

The M model, based on the N model, reformulates the repolarizing currents *I*_Kur_ and *I*_to_ using more recent experimental data. Furthermore, the electroneutral Na^+^ influx was removed, and an acetylcholine-activated K^+^ current *I*_KACh_ was added to simulate the effects of vagal stimulation. The AP and CaT look quite similar to those of the N model (Figures 1C,D). The aim of this model was to provide a more accurate description of the repolarization of a human atrial AP at different basic cycle lengths (BCLs) of stimulation and to investigate the effects of coupling between myocytes and fibroblasts.

The K model further extends the N and M models. It additionally includes a hyperpolarization-activated inward K^+^ current, *I*_f_. The aim of this model was to give a detailed description of the SR and Ca^2+^ release in the human atrial myocyte and to investigate the effects on Ca^2+^ dynamics and AP morphology. Therefore, intracellular Ca^2+^ and the SR incorporate a spatial dimension and are divided into four compartments (Figure 1B) due to the lack of T-tubules in atrial myocytes. The uptake and release unit of the first SR compartment interacts with the subspace below the membrane, whereas the other three units interact with the corresponding cytosolic Ca^2+^ compartments. Furthermore, SR Ca^2+^ release to the different compartments is described by a new, phenomenological Hodgkin–Huxley type formulation. Again, the resulting AP has a similar shape to that of the N model, although it has a low amplitude plateau phase as compared to the N and M models (Figure 1C). The CaT rises more slowly than that of all other models, but has a longer duration than that of the N and M models (Figure 1D).

The G model is the most recently published human atrial model and is based on a human ventricular model from the same group (Grandi et al., 2010), which in turn relies on the rabbit ventricular model of Shannon et al. (2004). The majority of the current formulations are similar to those found in the human ventricular model but with appropriate adaptation to human atrial data. In contrast to the other models presented, the concentration of Cl^−^, a background Cl^−^ current *I*_bCl_, and a Ca^2+^ activated Cl^−^ current *I*_Cl(Ca)_ are also taken into account. Furthermore, a plateau K^+^ channel *I*_Kp_ is included. For *I*_to_ and *I*_Kur_ currents, slightly adapted formulations of the M model were employed. The intracellular Ca^2+^ handling is identical to the rabbit ventricular model of Shannon et al. and includes a subsarcolemmal space and a junctional cleft between the *I*_CaL_ channels and the release unit. The CaT shows a slow rise as for the K model, but with a comparatively low amplitude and a slow decay to a high diastolic concentration. The AP appears roughly similar to the N, M, and K models, but with less pronounced overshoot. Furthermore, repolarization occurs in two distinct phases, resulting in a longer action potential duration (APD). This model was originally developed to analyze the differences between human atrial and ventricular electrophysiology with a focus on Ca^2+^ handling.

### 2.2. Modification of channel conductivities to represent cAF

Carefully modeling the physiology of the healthy human atrial myocyte as in the presented models is, of course, essential. However, in order to investigate the impact of dangerous atrial arrhythmias, models must be modified in order to account for disease-associated remodeling. The following section outlines how models can be modified to simulate the impact on cells' electrophysiology as occurs during chronic atrial fibrillation (cAF).

Wijffels et al. (1995) first suggested the principle that “AF begets AF,” asserting that the occurrence of AF itself leads to increased probability for cAF. The responsible electrophysiological mechanism, electrical remodeling, affects the expression of different ion channels as well as gap junctions, in concert with other influential processes (Schotten et al., 2011). To simulate cAF in the different models, this remodeling process was accounted for via direct modification of selected sarcolemmal ion channel conductivities. *I*_to_ and *I*_CaL_ were each decreased by 65%, *I*_Kur_ was decreased by 49% and *I*_K1_ increased by 110% (van Wagoner et al., 1997; Bosch et al., 1999; Dobrev et al., 2001). In tissue simulations, intracellular conductivity was reduced by 30% to account for gap junctional remodeling as shown in Seemann et al. (2010a).

In order to compare maximum current amplitudes of the different control sinus rhythm and cAF models, all models were investigated after 50 s pacing with a BCL of 1 s. The currents were normalized to the value of *I*_K1_ after 50 s clamped to a transmembrane voltage of −75 mV, as I_K1_ is the major open channel at resting phase, in order to better compare the current amplitudes of the different models.

### 2.3. Single-cell investigations

On the single-cell level, the models were benchmarked with respect to their electrophysiological properties as well as general model characteristics, such as long term stability. Stimulus amplitudes were adapted specifically for each model such that they were twice the threshold amplitude. Major baseline electrophysiological properties measured included amplitude, APD_50_, APD_90_, and resting membrane potential (RMP). These properties were sampled from the terminal AP after pacing for 50 s with a BCL of 1 s. This was chosen as a common starting point for the simulations with all models, since some of them did not exhibit a stable steady state.

In order to assess the long term stability of the models, several properties of interest were investigated. Each model was first paced for 20 min with a BCL of 1 s. The resulting AP amplitude, and APD at 50% and at 90% repolarization (APD_50_ and APD_90_, respectively) were measured for each paced cycle during this period. Furthermore, the RMP was observed over a period of 30 min. During the first 20 min, the models were not stimulated to make sure that the change of the RMP over time was minimal. During the last 10 min, models were paced with a BCL of 1 s, such that the transition of the models from a quiescent to a paced state could be observed.

The ability of each model to reproduce alternans was also assessed. At short BCLs, cardiac cells are not able to recover completely from the previous beat. This results in alternating AP patterns: a long AP and a short diastolic interval (DI) followed by a short AP and a longer DI. In order to investigate this behavior, each model was paced for 30 s with a BCL varying between 0.2 and 1 s and the resulting APD_50_ restitution was analyzed. Furthermore, the APD_50_ and [Ca^2+^]_i_ of 30 s pacing with a BCL of 0.25 s were then measured. APD_50_ was selected as an appropriate metric, as APD_90_ could not be computed in all cases since some APs did not reach 90% repolarization due to the short cycle length.

### 2.4. Tissue simulations

#### 2.4.1. 1D restitution curve

Steady state restitution curves of different electrophysiological properties were calculated in a 1D tissue strand (20 × 0.1 × 0.1 mm) with cubic voxels and homogeneous conductivity. For this purpose, 50 beats were calculated in a single-cell environment, so that models could first adapt to the different BCLs, which ranged between 0.2 and 1.0 s. As in the single-cell simulations, this common starting point was chosen, since some models did not exhibit a stable steady state. Afterward, stimulation from one side of the strand initiated five consecutive beats in the tissue, and properties were investigated following the last beat, provided all previous beats could initiate an AP. APD_90_ was recorded three-quarters of the distance down the strand. The conduction velocity (CV) was determined by dividing the distance between these measurement sites by the difference between activation times at the center of the first and the second halves of the tissue strand. The effective refractory period (ERP) was identified by applying an additional, premature stimulus at the same location as a first stimulus. The time between this initial stimulus and the first premature stimulus that could initiate an AP at the center of the second half of the strand was denoted as the ERP. Furthermore, the wavelength (WL), which can be defined as the distance traveled by an electrical impulse during the refractory period, was computed as the product of ERP and CV.

#### 2.4.2. 2D rotor initiation and tracking

As for 1D simulations, for 2D simulations 50 beats were first calculated in a single-cell environment in order to adapt all models to a BCL of 0.4 s. Four beats were then computed in an isotropic 2D tissue patch (100 × 100 × 0.1 mm) stimulated at the left side of the patch (see Figure 6). Following the fourth paced beat, a premature stimulus was simulated via stimulation applied to excited tissue at the patch's lower half. This cross-field (S1–S2) protocol with model-specific stimulation time S2 was used to initiate a rotor in the center of the patch. In case of rotor initiation success, the trajectories of the spiral cores were tracked using an algorithm based on that of Bray et al. (2001) which identifies phase singularities. The dominant frequency was also calculated via fast Fourier transform. For this purpose, a pseudo-ECG signal as described in Seemann et al. (2010a), was computed based on the intercellular current density distribution using two electrodes at 5 mm distance from the patch and 10 mm distance between each other in the center of the patch.

### 2.5. Numerical methods

All models were implemented in a modular C++ environment using a Rush–Larsen scheme for gating variables and a forward Euler scheme for the remaining ODEs. In the Grandi model, a fourth order Runge–Kutta scheme was used for the computation of intracellular and SR calcium concentrations as well as calsequestrin in order to ensure numerical stability. For both single-cell and tissue simulations, a time increment of 10 μs was generally used; however, the N, M, and K models required a time increment of 5 μs for 2D tissue simulations. Monodomain tissue simulations were performed using the parallel modular solver acCELLerate (Seemann et al., 2010b) wherein the finite difference method was applied. The 1D tissue strand (20 × 0.1 × 0.1 mm) and the 2D tissue patch (100 × 100 × 0.1 mm) both had cubic voxels of size 0.1 mm. The isotropic intracellular conductivity for 1D and 2D simulations was adapted specifically to each model to obtain a CV of ≈750 mm/s at a BCL of 1 s in the control case. The accuracy of the numerical scheme was tested in the 1D strand by variation of the time and space steps and measuring the CV. Therefore, the time step was decreased from 10 to 5 μ s, which resulted in an increase of the CV by less than 0.75% for all models. If the space step is increased from 0.1 to 0.2 mm, the CV is reduced by 2.5% at most, which is in the range of reported values (ten Tusscher et al., 2004; Clayton and Panfilov, 2008; Shajahan et al., 2009). For single-cell control and cAF simulations, the stimulus current amplitudes were adapted specifically for each model such that they were twice the threshold amplitude. In case of tissue simulations, the stimulus current amplitudes were 20% above threshold amplitude, which allowed for safe excitation propagation.

## 3. Results

### 3.1. Principal model characteristics

In initial single-cell simulations, basic model properties, such as CaT characteristics, RMP, and AP amplitude and duration were evaluated (Table 1). Corresponding experimental measurement data can be found in Table 3 for comparison. The AP amplitudes of the C, N, M, and K models lie roughly in the same range of measurement data of Bosch et al. (1999) of the right atrial appendage. Only the G model has a much smaller amplitude (around 30–40 mV smaller than the other models). In contrast, the RMP of the N, M, K, and G models is similar to measured data (Bosch et al., 1999; Christ et al., 2008) but more than 5 mV lower in the C model. Due to the spike-and-dome morphology of the AP of the C model, its APD_50_ is longer than that of the other models. The N and M models have similar APD_50_, due to their triangular shape, and the K model has the shortest APD_50_ due to a large peak amplitude and low plateau phase. The G model has a longer APD_50_ than the N, M, and K models, despite its relatively triangular morphology, as it does not present a significant overshoot. APD_90_ is longest in the G model and fits best to the measurement data of Christ et al. (2008) of the right atrial appendage. However, the C, N, and K models have a shorter APD_90_, in the range that has been measured in isolated cells by Bosch et al. (1999). The maximum upstroke velocity dV/dt_max_ of the APs is highest in the C model and comparable to data of Workman et al. (2001) measured at a BCL of 0.8 s. The values of the other models are significantly smaller; the measured upstroke velocity is even twice as fast as that of the G model.

**Table 1:** **Single-cell AP and calcium transient (CaT) properties of the different atrial models (BCL = 1 s)**.

**AP property**	**C**	**N**	**M**	**K**	**G**
Amplitude (mV)	110.11	116.14	119.14	120.77	81.38
RMP (mV)	−81.04	−74.15	−73.82	−76.13	−73.53
APD_50_ (ms)	165.16	29.55	29.77	20.98	125.30
APD_90_ (ms)	294.83	220.34	197.09	259.58	330.13
dV/dt_max_ (V/s)	186.58	149.77	160.66	168.54	92.50
[Ca^2+^]_i_ diastolic (μM)	0.115	0.065	0.065	0.136	0.208
CaT amplitude (μM)	0.649	1.235	1.227	0.496	0.257

The CaT properties of the models are compared to measurement data of Voigt et al. (2009) at a BCL of 2 s. The simulated values at this higher BCL (not shown) are similar to those specified in Table 1 at a BCL of 1 s. The C and K models present approximately the same diastolic intracellular calcium concentration as those measured (119.7 nM), whereas the N and M models compute lower and the G model higher diastolic concentrations. The amplitude of a measured CaT (344.9 nM) is best reproduced by the G and K models. In contrast, the C, N, and M model compute much higher amplitudes.

**Figure 2:**
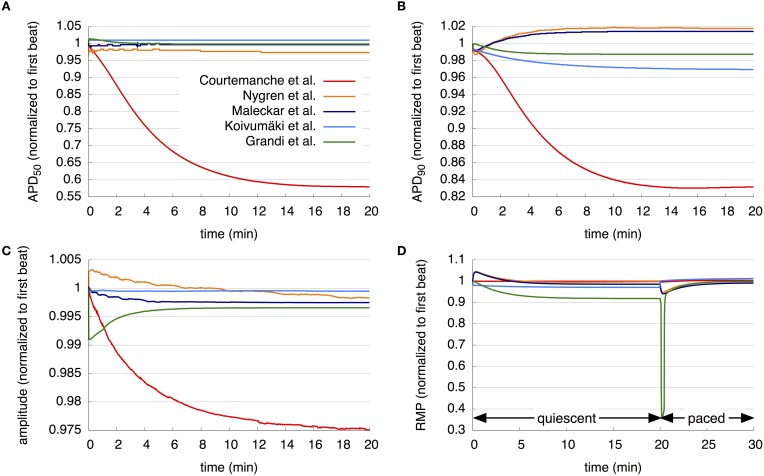
**Long term stability of the models. (A–C)** APD_50_, APD_90_, and AP amplitude over 20 min pacing with a BCL of 1 s. **(D)** RMP over 20 min without pacing followed by 10 min pacing with a BCL of 1 s. The resulting curves of the different models are compared in detail in the section 3.1.

Further simulations examined models' long term stability (Figure 2). The M, K, and G models show slight adaptation of APD_50_ during the first minutes of simulation at a BCL of 1 s, until a steady state close to the initial value is reached. In contrast, the C and N models reach steady state after a much longer simulation time. The APD_50_ in C has decreased by 42% over this period, whereas that of the N model by roughly 2.7%.

Assessing APD stability by observing APD_90_ over a simulation period of 20 min with a BCL of 1 s also reveals stark differences between models. N, M, and G models reach steady state at approximately 10 min, each with an APD_90_ close to the initial value. The APD_90_ of the K model decreases slightly over the period investigated to 96.9% of its initial value, but does not reach steady state in the time simulated. The C model shows a great variation in APD_90_ during the simulation time; APD_90_ first decreases during the first 16 min to 83% of its values during the first beat, and then again increases to 83.2% of its value during the initial beat after 20 min.

Under the same simulation regime, the AP amplitude of the N model increases, while that of the G model decreases, from the first to second beats by approximately 0.5% and 1%, respectively. Then, the amplitude of the N model continuously reduces to 99.8% of its initial value after 20 min, whereas that of the G model increases to a steady state of 99.8% after around 8 min. The M and K model results show only slight reduction in the AP amplitude during the first minutes of simulation, after which results are stable. AP amplitude of the C model reduces continuously to 97.5% after 20 min of simulation and does not reach steady state.

The development of the RMP over a period of 30 min was then examined. As detailed in section 2, during the first 20 min of simulation, cells are quiescent, followed by stimulation with a BCL of 1 s for 10 min. The C model shows almost no change in RMP during the quiescent phase, then a slight decrease after the initiation of pacing, followed by an increase. The N and M models show similar behavior during first 10 min: an increase and then a decrease in RMP, with a maximum in the first minute. After 10 min, the M model remains stable, whereas the RMP of the N model again increases. After the first stimulus, both the N and M models' RMP decreases and then increases again. The K model shows a reduction in RMP by almost 1.2% after the first timestep; the RMP continues to decrease slightly until it reaches a steady state after around 10 min. After the first stimulus, the RMP increases above the initial value and then continues to increase slightly. The RMP of the G model decreases for 10 min of quiescent simulation and then remains stable at 91.9% of its initial value. Following stimulation, the RMP in the G model rapidly declines to 35.9% of the initial value after 12 beats and then increases again to nearly 100% of its initial value.

The initial assessment of the models also revealed major differences in single-cell computing times. The C and N models show similar computing times [35.9 s vs. 37.4 s for the simulation of 1000 s at a BCL of 1 s on Mac OSX 10.7 (*Apple Inc., Cupertino, CA*) with a 2.7 GHz Intel Core i7 and 8 GB RAM without writing results to hard disk] and are the fastest. Computing time for the M model is increased 1.56-fold as compared to the C model, whereas that of the K and G models are 3.43 and 4.49 times longer, respectively.

### 3.2. Ability of the models to reproduce alternans and cAF-induced remodeling

**Figure 3:**
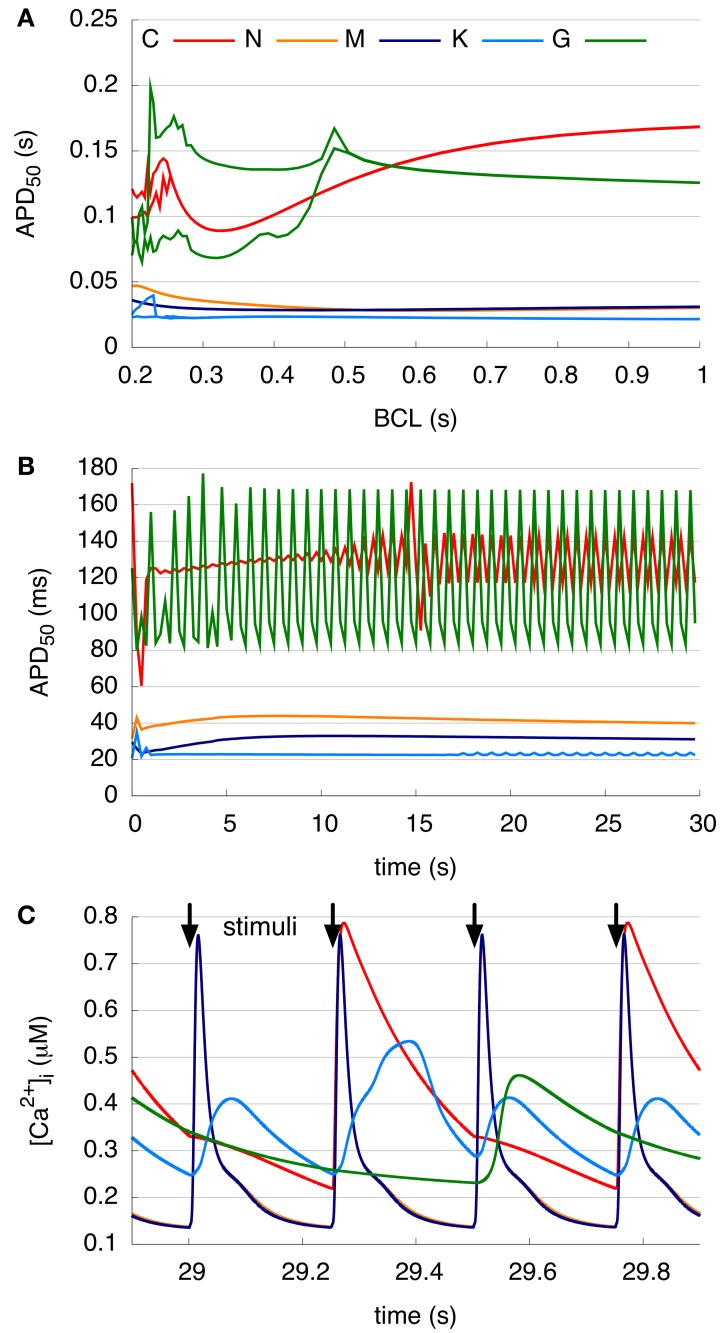
**Alternans at different pacing rates. (A)** Single-cell APD_50_ restitution curves resulting from 30 s pacing with a BCL between 0.2 and 1 s. C, K, and G models show bifurcation at short BCLs. **(B)** APD_50_ over 30 s rapid pacing with a BCL of 0.25 s. C model presents pronounced beat-to-beat alternans and G and K model produce a longer APD_50_ at every third beat. **(C)** Intracellular Ca^2+^ concentration between 29 and 30 s rapid pacing with a BCL of 0.25 s. Curves of N and M model calcium transient overlap. Peak of the Ca^2+^ transient of the C and G models visible at every second and third stimulus, respectively. K model initiates higher peak at every third stimulus, whereas N and M models cause a transient at every beat.

The ability of the models to represent the physiological phenomena of alternans is examined during 30 s of pacing (Figure 3). The APD_50_ restitution curve (Figure 3A) showed that no alternans were visible in either the N and M models, whereas the C and K models presented a bifurcation of the APD curves at a BCL of around 0.25 s and the G model already at 0.5 s. The APD_50_ at a BCL of 0.25 s (Figure 3B) of both the N and M models increases slightly up to 7 s of pacing, and then decreases without beat-to-beat alternans. The C model shows oscillations during the first 3 s as the model adapts to the short BCL, and then produced stable, pronounced beat-to-beat alternans of APD_50_. The K model presents small oscillations during the first second, and after a few seconds reverts to a nearly constant APD_50_. After 17 s, the APD_50_ of every third AP is longer than that of the others in the K model. After initial variations for 7 s, the G model also shows stable alternans with every third AP longer than the two previous beats. The peak value of the corresponding CaT (Figure 3C) is approximately 0.7–0.8 μM in the C, N, and M models and around 0.5 μM in the K and G models between 29 and 30 s of rapid pacing (BCL = 0.25 s). The C, K, and G models present higher diastolic calcium concentrations as compared to the two other models. The CaTs in the N and M models reveal a sharp peak and short duration for each stimulus. The C model results in CaTs of longer duration, sharp transitions, and a peak visible at every second stimulus. The K model shows two low-amplitude (0.4 μM) transients followed by a higher amplitude transient. In contrast, the G model shows just one long transient with a slow decrease at every third stimulus.

**Figure 4:**
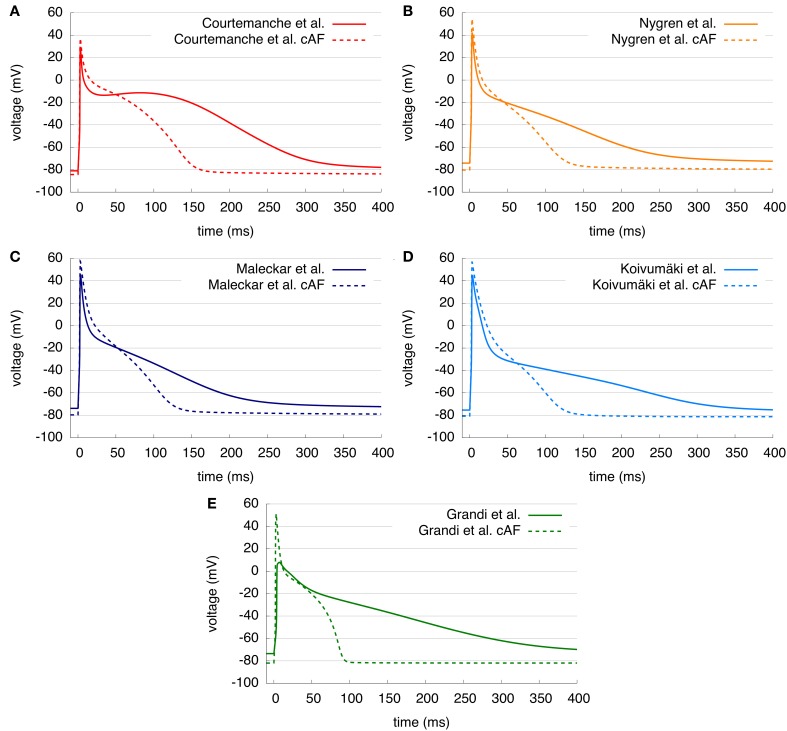
**(A–E)** Control and cAF APs of the five models. AP shape is triangular in C, N, M, and K models in case of cAF and APD duration is shortened in all models.

For the comparison of control and cAF APs, the models were modified to reproduce effects of electrical remodeling (changes outlined in section 2). Figure 4 shows the resulting APs of the five models. Figures 4A–E present a single AP of each model, following 50 s of pacing (at a BCL of 1 s). In the C, N, M, and K models, AP morphology appears triangulated in the cAF case, independent of the shape of the control AP. The G model reveals biphasic repolarization in the control as well as in the cAF case. Furthermore, upstroke velocity is increased in the G model in case of cAF and a pronounced overshoot can be observed. Table 2 presents the resulting AP amplitude, RMP, APD_90_, and dV/dt_max_ of the modified cAF models, which can be compared to measured values shown in Table 3. Only the C model produces an amplitude in the range of experimental data of Bosch et al. (1999), whereas the amplitude of the other models is 10–15 mV higher. The RMPs of the N, M, K, and G models are similar and lie in the range of experimental data, whereas the C model shows an increased RMP. The APD_50_ of the C model is between 35 and 45 ms longer than that of the other models. Similarly, the APD_90_ of the C model is also longest of all models but is shorter than the experimental values of Christ et al. (2008). The N, M, K, and G models better fit to the measurements of Bosch et al. (1999) and Workman et al. (2001). The upstroke velocity dV/dt_max_ of the N, M, and K models are similar, but lower than that of the C model, which fits best the experimental value of Workman et al. (2001). The G model has a more than 150 V/s faster upstroke velocity than the other models. The maximum current amplitudes of the different models during control sinus rhythm and cAF can be found in Table 4. For better comparison of the current amplitudes among the different models, they were normalized to the value of *I*_K1_ after 50 s clamped to a transmembrane voltage of −75 mV.

**Table 2:** **Single-cell AP properties of the modified cAF models (BCL = 1 s)**.

**AP property**	**C**	**N**	**M**	**K**	**G**
Amplitude (mV)	121.58	134.92	137.10	138.08	133.28
RMP (mV)	−84.34	−80.29	−79.65	−81.30	−81.99
APD_50_ (ms)	74.99	30.14	35.33	29.17	39.54
APD_90_ (ms)	143.87	115.13	115.59	109.21	85.70
dV/dt_max_ (V/s)	211.38	174.96	182.47	179.26	359.53

**Table 3:** **Experimental values (where available) of control sinus rhythm and cAF AP properties of Bosch et al. (1999), Workman et al. (2001), and Christ et al. (2008)**.

**AP property**		**Bosch et al.**	**Workman et al.**	**Christ et al.**
Amplitude	Control	116±3		
(mV)	cAF	120±2		
RMP	Control	−76.3±2.2	−76.9±2.1	−75.0±0.4
(mV)	cAF	−78.9±2.9	≈−77	−78.9±1.1
APD_90_	Control	255±45	209±22	≈344
(ms)	cAF	104±9	95±12	287±16
dV/dt_max_	Control		203±11	
(V/s)	cAF		231±16	

Data of Workman et al. was obtained at a BCL of 0.8 s.

**Table 4:** **Maximum current amplitudes of the currents common to all models^a^ during control sinus rhythm and cAF after 50 s pacing with a BCL of 1 s**.

**Current**	**C**		**N**		**M**		**K**		**G**	
	**Control**	**cAF**	**Control**	**cAF**	**Control**	**cAF**	**Control**	**cAF**	**Control**	**cAF**
*I*_Na_	−440.15	−427.77	−317.39	−328.65	−347.84	−370.27	−332.62	−375.75	−564.11	−1749.59
*I*_bNa_	−0.23	−0.23	−0.41	−0.43	−0.41	−0.42	−0.40	−0.42	−0.51	−0.54
*I*_CaL_	−9.88	−3.62	−10.48	−3.57	−9.87	−3.15	−19.50	−5.09	−47.56	−18.77
*I*_bCa_	−0.55	−0.57	−0.77	−0.81	−0.76	−0.79	−0.78	−0.80	−0.68	−0.71
*I*_NCX,max_	1.33	1.48	2.81	3.61	2.91	3.66	0.69	0.91	0.16	0.70
*I*_NCX,min_	−0.76	−0.78	−1.72	−0.95	−1.55	−0.88	−0.76	−0.83	−5.02	−1.62
*I*_NKA_	0.54	0.54	0.89	0.90	0.86	0.86	0.89	0.92	1.64	1.67
*I*_PMCA_	0.38	0.27	0.16	0.15	0.16	0.15	0.06	0.05	0.26	0.14
*I*_to_	23.31	10.69	19.59	9.28	23.00	10.67	24.16	10.61	30.39	25.82
*I*_Kur_	7.03	5.45	13.67	8.15	11.34	6.67	12.66	7.24	16.29	15.52
*I*_Kr_	0.55	0.50	0.07	0.10	0.08	0.12	0.09	0.13	0.07	0.53
*I*_Ks_	0.21	0.09	4.98	6.05	0.04	0.07	0.05	0.07	0.01	3.34E-3
*I*_K1_	1.17	2.46	1.24	2.60	1.29	2.69	1.29	2.71	1.33	15.91

The currents are normalized to the value of I_K1_ after 50 s clamped to a transmembrane voltage of −75 mV.

a Model specific currents:

M model: I_KACh_ = 6.09E-11 (6.30E-11); K model: I_f_ = 0.20 (0.33); and G model: I_Kp_ = 2.18E − 3 (1.17), I_Cl(Ca)_ = 0.80 (0.12), I_bCl_ = 3.64 (5.92).

### 3.3. Restitution properties in 1D tissue

**Figure 5:**
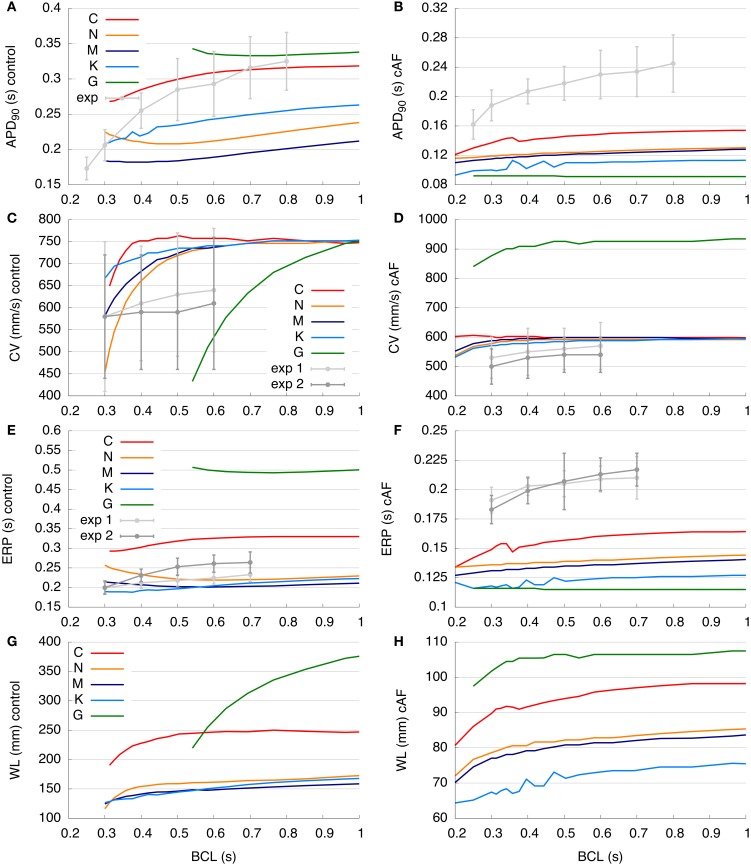
**Restitution curves of the control and cAF models. (A,B)** APD_90_ restitution compared to experimental data of Franz (1997). **(C,D)** Simulated and measured (Feld et al., 1997) CV restitution (exp1: right atrial free wall, exp2: septum). **(E,F)** ERP restitution compared to experimental data of Yu et al. (1999) (exp1: right atrial appendage, exp2: distal coronary sinus). **(G,H)** Simulated WL restitution. Panels **(C,E)**, and **(G)** modified according to Dössel et al. (2012). Restitution curves are described in detail in section 3.3.

Various dynamic properties in tissue of all models are compared to experiments and are presented in Figure 5. In Figures 5A,B, the control and cAF models, respectively, are compared to the experimental data of Franz (1997). Models' APD_90_ restitution at BCLs from 0.2 to 1 s are presented. Shown in Figure 5A (control), the C model could not initiate APs at every beat in tissue simulations at a BCL of 0.31 s or lower; the G model exhibited similar behavior with a limiting BCL of 0.54 s. The other models could still be evaluated at BCL of 0.3 s or smaller in tissue simulations. Both the C and G model results lie within the range of experimental data of APD_90_. The C model shows a similar slope as compared to experiment, whereas the other models are lower than experimental data at BCLs higher than 0.3 s. Furthermore, the slopes of APD_90_ restitution curves do not match experimental data; the N, M, and G model even reveal increasing APD_90_ as BCL decreases. In Figure 5B, the cAF versions of the models are presented. Stimulation of the C, N, M, and K models in tissue at BCLs down to 0.2 s resulted in unique APs, whereas the G model could be stimulated down to a BCL of 0.25 s. The APD_90_ of all cAF models is below that of available experimental data. The slopes of the simulated restitution curves are also smaller than those of the experimentally measured curve; for instance, the G model shows nearly a constant APD over the investigated BCLs.

The control and cAF models, respectively, are compared to the experimental data of Feld et al. (1997) in Figures 5C,D. Models' CV restitution curves at BCLs from 0.2 to 1 s are presented there. In Figure 5C, describing the control case, the simulated values of CV in C, N, M, and K models are above that of mean experimental data. However, these models' CV are in the range of standard deviation of experimental data (simulated values around 750 mm/s at high BCLs), as intracellular conductivity in the 1D myocyte strand (described in section 2) was adapted to obtain this value at a BCL of 1 s. CV measured in experiments decreases slightly with decreasing BCL, whereas CV in the C model slightly increases at BCL of 0.5 s as compared to a BCL of 0.6 s (indicating supernormal conduction). The CVs of both the N and M models decrease markedly from a BCL of 0.4 s to a BCL of 0.3 s as compared to experiments, which present a much more gradual decrease in CV with increased pacing frequency. The K model reveals similar changes with respect to restitution slope as compared to experimentally measured CV. The control G model shows the most rapid decrease of CV from a BCL of 1 s toward a BCL of 0.6 s. At a BCL of 0.4 s, which is the BCL used for the initiation of a rotor in the 2D patch, the CVs of the models were: C (751.9 mm/s), N (657.9 mm/s), M (680.2 mm/s), K (714.3 mm/s), and the control G model could not be analyzed at BCLs shorter than 0.54 s. In Figure 5D, the CV restitutions of the cAF versions of the models are presented. In general, the C, N, M, and K models are roughly in the range of available experimental data; only the G model reveals a much higher CV as compared to experimental measurements. As for the control case, the CV of the C model increases as pacing moves toward lower BCLs (supernormal conduction), although experimentally measured CV decreases. The N, M, K, and G cAF models reproduce the slope of the CV restitution curve well. Using the same BCL = 0.4 s as in the 2D rotor simulations, the CVs obtained were: C (602.4 mm/s), N (588.2 mm/s), M (595.2 mm/s), K (578.0 mm/s), and G (909.1 mm/s).

In Figures 5E,F, the ERP restitution of control and cAF models, respectively, from BCLs from 0.2 to 1 s are compared to the experimental data of Yu et al. (1999). Figure 5E examines the behavior of control models, wherein the values of N, M, and K models are roughly in the range of experimental data. The ERP of the G model is more than 200 ms higher than available measurement data, while values of the C model ERP restitution are around 50 ms higher than experiments. In experimental data, ERP decreases with decreasing BCL, and the ERP restitution slope of the C model fits best to that of experimental data. In contrast, the ERP of N, M, K, and G models increases even as the BCL is decreased. The ERPs at a BCL of 0.4 s were: C (302 ms), N (234 ms), M (207 ms), K (192 ms), and G could not be analyzed. When the ERP of all cAF models is compared to available experimental data (Figure 5F), it is evident that model results are of much lower magnitude (as in case of APD_90_, Figure 5B). In this case, the slopes of all simulated restitution curves are too low as compared to experimental measurements, and the K and G models reveal slightly increasing ERP for high pacing frequency (BCL of 0.2 s). At a BCL of 0.4 s, following values could be obtained: C (152 ms), N (137 ms), M (133 ms), K (123 ms), and G (116 ms).

In Figures 5G,H, the WL restitution of control and cAF models, respectively, is presented from BCLs from 0.2 to 1.0 s (no experimental data was available for comparison). Figure 5G displays results for the control models; the G model has the longest WL (up to 375 mm at the highest BCL of 1.0 s). In comparison, the C model has a far shorter WL (maximally 250 mm and nearly constant for BCLs longer than 0.5 s). The N, M, and K models all feature WLs in the same range (around 150–170 mm at the highest BCL computed). The WL slopes of both the C and N models are comparably high at low BCLs, whereas the slopes of the M and K models are smaller at low BCLs, indicating a lesser sensitivity to rate. In contrast to the C model, which reveals a local maximum in WL around a BCL of 0.7 s, the WLs of the N, M, K, and G models each decrease from the highest to lowest BCL. WL in the G model reveals the greatest sensitivity to change in BCL. The WLs at the same BCL of 0.4 s as during the 2D simulations were: C (227.1 mm), N (153.9 mm), M (140.8 mm), K (137.1 mm), and G could not be analyzed. When the WLs of all cAF models are compared (Figure 5H), it is again seen that the WL of the G model is longer than that of all other models. The WL of the K model is the shortest of all models. Although the WL decreases as BCL is decreased and the slope of these WL restitution curves is similar for all models, only the G model reveals nearly constant WL at longer BCLs. Nevertheless, a decrease in the sensitivity of WL to rate is evident for all models in case of cAF. At a BCL of 0.4 s, the resulting WLs were: C (91.6 mm), N (80.6 mm), M (79.2 mm), K (71.1 mm), and G (105.4 mm).

### 3.4. Dynamics of simulated arrhythmia in 2D

**Figure 6:**
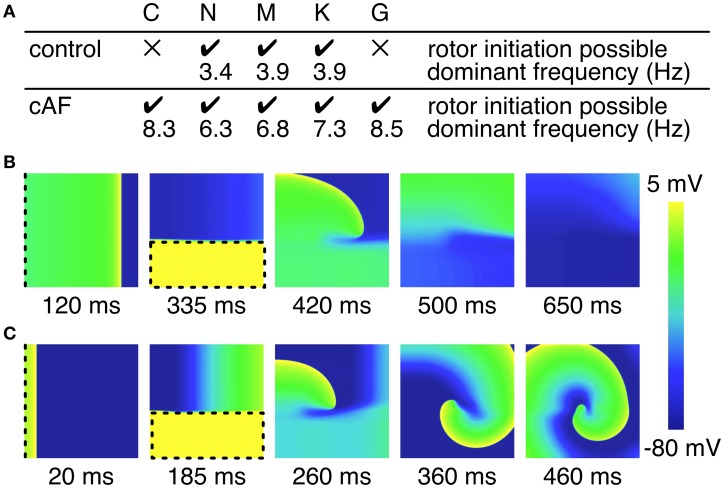
**Initiation of rotors in 2D tissue patch. (A)** Overview of rotor initiation success and corresponding dominant frequency. Control C and G model failed to initiate a rotor in the 2D patch (100 × 100 × 0.1 mm). Higher dominant frequencies could be observed in case of cAF. **(B)** Screen shots of failed rotor initiation in the control C model, where the WL was too long related to the patch size. **(C)** Successful rotor initiation in the cAF C model. Dashed lines indicate stimulus sites and area, respectively.

The facility with which a single rotor can be initiated in a 2D atrial tissue patch employing control or cAF versions of each of the models was examined (Figure 6). For control models, only the N, M, and K models permitted the initiation of a rotor, whereas the atrial tissue patch including C and G models failed to produce a reentrant circuit when subjected to an identical protocol. The dominant frequency extracted from the pseudo-ECG was comparatively low in all models, with frequency slightly increasing from the N to the M and K models from 3.4 to 3.9 Hz. In comparison, a rotor could be induced using all of the cAF models. The dominant frequency of the C and G models was much higher than that of the N, M, and K models (average 8.4 Hz as compared to an average of 6.8 Hz, respectively). Experimentally measured dominant frequencies generally lie within a range of 4–9 Hz with higher frequencies indicating more persistent forms of AF (Ng and Goldberger, 2007).

**Figure 7:**
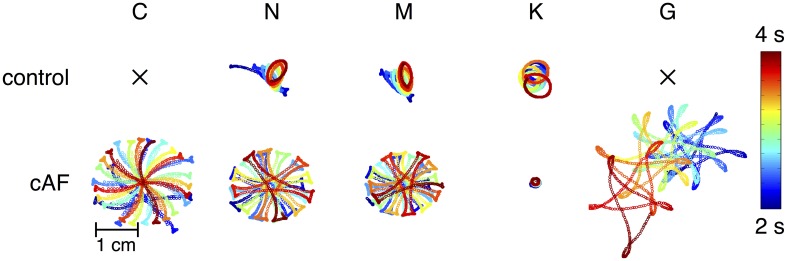
**Mapping of rotor center trajectories after initiation of reentrant circuit in 2D tissue patch using the control and cAF models.** Control C and G model failed to initiate a rotor in the 2D patch. N, M, and K models show ellipsoidal trajectory in the control case. The K model shows a stable circular trajectory in case of cAF, whereas rotors of the other models present a meandering star-shaped trajectory occupying more space.

In order to better understand the dynamics of rotors induced using different versions of the models, trajectories of rotor centers in control and cAF versions of each of the models are presented in Figure 7. Rotor centers were tracked between 2 and 4 s during simulation. In the control versions of the C and G models, no rotor could be initiated, whereas in the cAF versions, induced rotors revealed regularly meandering wave tips with slightly curved, star-shaped trajectories. The trajectory in the simulations employing the cAF G model occupied the largest area of the atrial patch. The N and M models show similar trajectories in both control and cAF versions. In the control case, trajectories had the shape of small ellipsoids, whereas in the case of the cAF models, these took the shape of a star and occupied a larger area than in control. The K model shows an elliptical trajectory in the control case and occupies a similar area as compared to those of the N and M models. However, in simulations employing the cAF version of the K model, the rotor center remains stable on a markedly small circle.

## 4. Discussion

The results presented in the previous section jointly characterize and compare five published human atrial cell models (C, N, M, K, and G). The primary goal was to clearly establish, for the first time, the principal characteristics and model differences with respect to (1) model long-term stability, (2) the ability to reproduce alternans and AF-induced remodeling, (3) restitution properties in 1D tissue and (4) dynamics of simulated arrhythmia in 2D.

### 4.1. Comparative findings

Stark differences in AP morphology are to be expected in models of the human atrial myocyte, as a wealth of data reveals divergent AP morphologies from different atrial cell types (Gelband et al., 1972; Wang et al., 1993), from cells dissociated from different regions of the human atria (Caballero et al., 2010), and those remodeled during disease (van Wagoner et al., 1997; Bosch et al., 1999; Dobrev et al., 2001). In essence, it may be that no single biophysical representation of the human atrial myocyte can reasonably be expected to reflect the inherent diversity in cell phenotype present in the human atria.

Although essential for their robust usage in both cell and tissue level investigation of the human atria, the stability of dynamic properties under typical protocols had not been presented previously. APD_50_ for N, M, K, and G models reaches steady-state after the first minutes of pacing (Figure 2), while the C model reaches steady state at approximately 20 min with the APD_50_ decreased by approximately 42% over this period. APD_90_ in the N, M, and G models reaches steady state after approximately 10 min, each with an APD_90_ close to the initial value, while APD_90_ in the K and C models does not reach steady state in the time simulated. The APD_90_ of the K model decreases slightly over the period investigated, while the C model shows a great variation during simulation. Although behaviors differ slightly with pacing, the AP amplitude of the M, K, and G models reaches steady state after the first minutes of simulation, while the AP amplitude of the C and N models does not reach steady state during 20 min of simulation. In the quiescent phase, all models appear to reach a steady state after 10 min with the exception of N, which is still drifting slightly. The highlighted differences in stability properties should be considered by the user when selecting a model for a given application. For example, the C model might not be suitable for in silico experiments, in which pacing needs to continue for a longer period.

The physiological phenomena of alternans (see Figure 3) is closely linked to atrial rhythm disturbances, and thus the ability to reproduce this phenomenon in the *in silico* cell may be desirable for some applications. No alternans were visible in the N and M models in single-cell simulations. In contrast, the C, K, and G model produced alternans, although with diverging dynamics and amplitude. It is clear that the ability of the models to reproduce alternans is dependent upon intracellular calcium concentration [Ca^2+^]_i_ (Figure 3C): the CaTs of the C and K models reveal calcium alternans corresponding to the alternans in APD_50_. The underlying mechanisms for alternans in these models is also diverging, in that the SR calcium release in C model has phenomenological dependence on membrane voltage, whereas the K and G model rely on a more physiological description of calcium-induced calcium release.

Figure 4 compared AP morphology for both standard and cAF versions of models. AP amplitude is increased in all models; the C model most closely matches experimental data. RMP became more negative in the case of cAF in all models as predicted by experimental data; again the C model compares most favorably to available data. APD_90_ was reduced in models and in the experimental data; in this case, no model clearly distinguishes itself as more accurate in terms of available data. All models appear to reproduce cAF characteristics, as an example of remodeling in disease, rather coherently. However, AP morphology of the G model appears to depend quite strongly on RMP and the amplitude of stimulus current. Therefore, the more negative RMP in the G model due to cAF remodeling causes a higher upstroke velocity and overshoot than in the control case.

Various dynamic properties of control and cAF models in tissue were also compared to experiments and presented in Figure 5. Control model dynamic properties reveal differences when compared to experimental data; APD_90_ restitution slopes (Figure 5A) of all models appear too flat as compared to experiments, model CV restitution magnitude is in the range of experiments, but appears much too steep at fast pacing rates as compared to experiments (Figure 5C), model ERP restitution is generally flatter than seen in experiments, though models diverge in behavior in this case (Figure 5E). cAF model dynamic properties also reveals difference from experimental measurements. APD_90_ restitution slope (Figure 5B) better matches experimental data but now is short as compared to experiments. Model CV restitution is still close to measured experimental magnitudes (with the exception of G model), though restitution is appropriately flattened (Figure 5D). The higher CV in case of cAF is caused by the higher upstroke velocity of the AP in the G model. Model ERP restitution slopes are similar to experiments, though magnitudes are diverse and model results are much smaller than measurements (Figure 5F). No experimental data is available for comparison in the case of WL restitution; however, it can be noted that model results for both control and cAF models are diverse, implying potential differences in how these models may reproduce dynamic phenomena at the tissue-level, including inducibility and rotor dynamics. This is indeed the case (Figure 6); for control models, only three of five models produce a reentrant circuit when subjected to an identical protocol while, in comparison, a rotor could be induced using all of the cAF models. In control models, when rotors could be initiated, trajectories generally assumed the shape of small ellipsoids, though the area described by the trajectory differed based on the model in question. In the cAF model versions, there was considerably more diversity. Rotor trajectories took the shape of a star and occupied a larger area than in control (N and M), revealed regularly meandering wave tips with slightly curved, star-shaped trajectories (C and G), or remained stable on a markedly small circle (K). This example succinctly illustrates the point that relatively small differences between human atrial myocyte models may indeed result in fairly large differences in emergent biophysical behaviors in tissue simulations.

### 4.2. Previous benchmarking and model comparison

The present work, although the most comprehensive, is not the first attempt at comparing published models of human atrial myocyte cell electrophysiology. In the absence of newer models, most previous work (Nygren et al., 2001; Cherry and Evans, 2008; Cherry et al., 2008) compared only the C and N models with the goal of clarifying the properties and biophysical predictions of each. For instance, as indicated in Results here, the C and N models diverge in terms of AP morphology and general dynamic properties. Nygren et al. (2001) asserted that the primary difference between the models lies in the assumed AP shape, and the corresponding sizes of the underlying ionic currents. For instance, the C model AP morphology was said to rely primarily on *I*_Kr_ and *I*_Ks_ for repolarization, while the N model depended on *I*_sus_ (*I*_Kur_) for repolarization reserve, and the rate-dependent properties of C as compared to N models based on the larger L-type calcium current in the former. Although such a detailed ionic analysis is not included here, it can be said that the M and K models represent progressive steps away from the inheritance of the N model with respect to morphological differences, as M updates several potassium currents, including *I*_to_ and *I*_Kur_, while K steps further to update intracellular Ca^2+^ dynamics in an atrial-specific fashion. The G model has another basis entirely (Shannon et al., 2004), but includes a detailed refitting of repolarization currents (*I*_Ks_, *I*_Kr_, and *I*_K1_) based on newly available experimental data from human atrial myocytes.

Later work (Cherry and Evans, 2008; Cherry et al., 2008) expands the comparison between the vastly different C and N models to include a systematic analysis of the models' dynamic properties in tissue (one-dimensional cables and rings, two-dimensional sheets, and a realistic three-dimensional human atrial geometry). The authors observe that the C and N models adapted divergently to changes in stimulation rate; the C model revealed the greatest adaptation in APD with rate (also seen here, Figure 5A). It was also observed in (Cherry and Evans, 2008) that reentrant wave dynamics differed, as C exhibited “frequent, transient wave breaks,” whereas the N model produced stable spiral waves in 2D tissue. Cherry and Evans observed this transient wave break in the control “CM” model using tissue patches up to 30 × 30 cm. In our patch simulations (10 × 10 cm), we observed no such behavior in the C model (see Figure 7), likely due to a larger CV and therefore a prohibitively long WL for the tissue patch size used in the present study. As the cycle length at which the tissue patch is paced previous to cross-field stimulation is not specified, it is difficult to ascertain whether *pre-shock* state of the tissue may have been a factor. However, a rotor could also be initiated in the tissue patch used in this study, if the gap junction conductance is sufficiently reduced (as e.g., shown in Majumder et al., 2011) according to the critical mass hypothesis (Qu, 2006; Panfilov and Pertsov, 2001). As in Figure 7, Cherry and Evans also observed a “stable spiral wave” without wave breaks in the “CM-AF” model. Thus there may be agreement between previous work and the results found here: in cAF remodeled tissues, both C and N models evince stable spiral waves, while in the control case, N supports a stable rotor, whereas the C model does not.

In a recent work of our group (Dössel et al., 2012), an overview of modeling human atrial electrophysiology and the five models benchmarked in this study is given. The different single-cell AP morphologies and durations of the control models are described: the spike-and-dome morphology of the C model and the triangular shapes of the N, M, K, and G models. Furthermore, differences in the restitution curves of the single-cell APD_90_ and the 1D strand CV, ERP, and WL are compared as in the present study. The N, M, and K models showed similar restitution properties, whereas the C and G models showed longer ERPs and WLs and steeper restitution curves toward shorter BCLs. Additionally, some exemplary applications of the models, e.g., modifications for the simulation of AF or atrial heterogeneity, described in literature are reviewed. However, a detailed comparison of general model properties, such as long term stability, alternans or the initiation of rotors in a 2D patch, as well as a comparison to experimental data is missing. Furthermore, an analysis of altered model characteristics due to e.g., cAF was not carried out in this work.

### 4.3. Experimental electrophysiology and the computational modeling

The quality and relevance of mathematical models are directly tied to the ability to observe, fit, and validate them in terms of empirical observations (Niederer and Smith, 2012). All models examined here have depended on measurement data relating to electrophysiological experiments on cardiac myocytes, predominantly atrial. Notably, much of the data used to first parameterize and then validate the models did not arise from experiments on human cells. Although comparative physiological study has offered significant information thus far, more data from human ion channels, cells and tissues is necessary, as species-level variation in electrophysiological properties can be significant. In addition, control data currently available from human sources often arises from hearts which suffer from coronary artery disease or pathology of unknown/unregistered etiology and expression, which may introduce uncharted remodeled properties to tissue deemed as healthy, as well as heterogeneity in sample quality.

There currently also exists a lack of understanding in the human atria in terms of scales: how does an emergent ion channel property affect the cellular-level properties and flow upward to influence dynamics at the tissue and organ levels? Mechanistic understanding between spatial and temporal levels is an arena in which computational models can contribute immensely in cardiac research (Zhou et al., 2009; Moreno et al., 2011; Pueyo et al., 2011; Sarkar et al., 2012). However, the challenge of multiscale research is complicated further as data at the channel level may have either or both transfected or natively occurring channels as origin. The role that such a difference in provenance may engender is thus far completely unclear. In addition, the modifying subunits of divergent channel isoforms have been shown to strongly influence dynamic channel properties and currents (Pourrier et al., 2003; Abbott et al., 2007; Patino and Isom, 2010; Olesen et al., 2012). However, key ion channels are often modeled mathematically as based on alpha subunit data only, limiting the ability of models to represent de facto physiology and their utility in uncovering emergent multiscale behaviors.

It is important to note that the relative scarcity of published data for validation make comparison of dynamic model properties to experiments in the present context rather difficult. These models are generalized in the sense that each is theoretically designed to represent a typical cell, but must then be directly compared to measurements from just a single-cell or a limited subset of cells. Because divergent physiology between individuals, regions, and preparations can result in very different experimental measurements of a human atrial myocyte, it may be that the particular experimental data available for comparison is essentially inappropriate for this purpose and results in model comparisons which are of limited utility.

### 4.4. Implications

The results presented here may provide useful information as to when each of the respective models might be appropriate for a particular study. In consideration of the control, healthy models, for instance, the M and G models are appropriate for studies which aim to include effects of vagal innervation, as *I*_KACh_ is included in the M model and the G model offers an optional formulation for this current, while the K and G models are a clear choice for any study interested in Ca^2+^ dynamics, despite the higher computational load. The N, M, K, models do offer long-term stability with stable transitions from quiescent to stimulated states, which closely represent the physiologically observed behaviors in single-cell experiments. K and C models best reflect APD_90_, while CV and ERP are badly matched by all models when considering dynamic properties observed in experiments. When considering the cAF models as compared to available experimental data, APD_90_ restitution appears to be too flat for all models, and APD is, in general, too low as compared to the experiment, while CV is better matched for all models. The simulated ERPs are too low for all models benchmarked as compared to the experiment. However, this comparison relates to only one set of experimental data; obtaining further dynamic measurements in healthy and electrophysiologically remodeled tissue is needed for further model validation.

It is a bit unclear as to what model might be the most desirable in terms of tissue dynamics and the study of arrhythmia mechanisms. For the initiation of rotors in control (non-remodeled) models, the N, M, and K models may be indicated, as C and G were non-inducible with the chosen simulation set-up (reflecting average tissue size in the human atria). For cAF models, however, arrhythmia was inducible using all models, but with divergent spiral wave dynamics, implying that the chosen model should depend specifically on the application, e.g., if wave pinning is key, the K model might be the most appropriate choice (Figure 6). The C model, however, is uniquely consistently able to reproduce stable beat-to-beat alternans (Figure 3) for tissue-level simulation. A caveat may be that the physiological underpinnings of alternans may be diverse, and not always relate to the underlying dynamics permitting alternans in the C model. Therefore, there likely exist several etiologies of tissue instability and dispersion for which current ionic models cannot account.

The models benchmarked here each represent a unique instance of human atrial myocyte electrophysiology. However, this diversity does not necessarily indicate particular shortcoming on behalf of any of the models. The differences in properties detailed previously may reflect the inherent heterogeneity in human atrial myocytes and even regional differences in electrophysiological properties in the human atria. Indeed, previous computational work from our lab and others (Seemann et al., 2006; Aslanidi et al., 2011) has employed regionally-specific modifications in ion channel conductances to reflect AP heterogeneity in the atria. The challenge of inherent heterogeneity in non-diseased tissue also calls to question approaches for disease modeling: the relatively simple, fixed alteration of ion channel conductances to reflect electrical remodeling in cAF, as effected in this study, is likely inadequate. Especially the investigation of intracellular calcium dynamics, whose changes due to cAF were neglected in this work, would lead to weak conclusions. Future development of models of this type in health and disease may very well lie in dynamic parameter fitting and adjustment, facilitated by newly applied and novel methods (Tondel et al., 2011; Sarkar et al., 2012).

### Conflict of interest statement

The authors declare that the research was conducted in the absence of any commercial or financial relationships that could be construed as a potential conflict of interest.
